# The impact of *ALDH7A1* variants in oral cancer development and prognosis

**DOI:** 10.18632/aging.204099

**Published:** 2022-05-25

**Authors:** Hsueh-Ju Lu, Chun-Yi Chuang, Mu-Kuan Chen, Chun-Wen Su, Wei-En Yang, Chia-Ming Yeh, Kuan-Ming Lai, Chih-Hsin Tang, Chiao-Wen Lin, Shun-Fa Yang

**Affiliations:** 1 Division of Hematology and Oncology, Department of Internal Medicine, Chung Shan Medical University Hospital, Taichung, Taiwan; 2 School of Medicine, Chung Shan Medical University, Taichung, Taiwan; 3 Department of Otolaryngology, Chung Shan Medical University Hospital, Taichung, Taiwan; 4 Department of Otorhinolaryngology-Head and Neck Surgery, Changhua Christian Hospital, Changhua, Taiwan; 5 Oral Cancer Research Center, Changhua Christian Hospital, Changhua, Taiwan; 6 Institute of Medicine, Chung Shan Medical University, Taichung, Taiwan; 7 Department of Medical Research, Chung Shan Medical University Hospital, Taichung, Taiwan; 8 Division of Hematology and Oncology, Department of Medicine, Changhua Christian Hospital, Changhua, Taiwan; 9 School of Medicine, China Medical University, Taichung, Taiwan; 10 Chinese Medicine Research Center, China Medical University, Taichung, Taiwan; 11 Department of Medical Laboratory Science and Biotechnology, College of Medical and Health Science, Asia University, Taichung, Taiwan; 12 Institute of Oral Sciences, Chung Shan Medical University, Taichung, Taiwan; 13 Department of Dentistry, Chung Shan Medical University Hospital, Taichung, Taiwan

**Keywords:** ALDH7A1, polymorphism, oral cancer, survival

## Abstract

The gene encoding aldehyde dehydrogenase 7 family member A1 (ALDH7A1) has been associated with the development and prognosis in multiple cancers; however, the role of *ALDH7A1* polymorphisms in oral cancer remains unknown. For this purpose, the influences of *ALDH7A1* rs13182402 and rs12659017 on oral cancer development and prognosis were analyzed. Our resulted showed that *ALDH7A1* rs13182402 genotype had less pathologic nodal metastasis among betel quid chewer. *ALDH7A1* rs13182402 also corresponded to higher expressions in upper aerodigestive mucosa, whole blood, the musculoskeletal system and oral cancer tissues than did the *ALDH7A1* wild type. Furthermore, ALDH7A1 overexpression in oral cancer cells increased *in vitro* migration, whereas its silencing reduced cell migration. Conversely, ALDH7A1 expression in tumor tissues and in patients with advanced disease was lower than that in normal tissues and in patients with early-stage disease. When the patients were classified into ALDH7A1-high and -low-expression groups, the high-ALDH7A1 group had superior outcomes in progression-free survival than the low-ALDH7A1 group (5-year survival of 58.7% vs. 48.0%, *P* = 0.048) did. In conclusion, patients with high ALDH7A1 expression might, however, have more favorable prognoses than those with low ALDH7A1 expression have.

## INTRODUCTION

The aldehyde dehydrogenase (ALDH) superfamily, encoded by ALDH genes, is crucial for metabolizing physiological and pathophysiological aldehydes [1]. *ALDH* polymorphisms or mutations reduce the activities of ALDH and increase acetaldehyde, which is toxic, mutagenic, and carcinogenic. Acetaldehyde also results in deoxyribonucleic acid (DNA) adducts, inhibited DNA repair, and DNA methylation [2]. As many as 19 *ALDH* genes have been identified within the human genome, and several diseases have been proven to be associated with *ALDH* mutations [3]. However, the role of individual ALDH genes in cancer development and prognosis has been a subject of controversial discussion.

ALDH 7 family member A1 (ALDH7A1), a member of the ALDH superfamily, is the enzyme encoded by *ALDH7A1* [4]. Several studies have proven the relationship between *ALDH7A1* mutations and pyridoxine-dependent seizures in children [5]. *ALDH7A1* dysfunctions have also been associated with other disorders, such as osteoporosis and Huntington’s disease, as well as with the mechanism of intracellular transport [6–9]. However, the role of *ALDH7A1* in cancer development and prognosis has remained unclear. The roles of the different *ALDH7A1* polymorphisms may vary, moreover, on account of different allele mutations and cancer types [10, 11].

Oral cancer, a subgroup of head and neck squamous cell carcinoma (HNSCC), is the sixth most common cancer globally and the fourth most common cancer in Taiwanese men [12, 13]. Although several innovative treatments that are effective in prolonging survival have been developed and approved [14, 15], over 50% of patients using those treatment agents still progressed to recurrent metastatic status, and only 20%–30% of them experienced long-term survival [16, 17]. With the development of next-generation sequencing, applying genetic information to cancer risk prediction, diagnosis, and treatment has become more feasible [18–20]. The role of genetic polymorphism in cancer development and progression is also critical.

In this study, we enrolled patients diagnosed as having oral cancer and healthy controls as participants. The *ALDH7A1* single-nucleotide polymorphisms (SNPs) of these participants were retrospectively tested. The effects of *ALDH7A1* polymorphism were compared for all participants, those who habitually chewed betel quid, and those who did not chew betel quid. Furthermore, published databases, such as Genotype-Tissue Expression (GTEx) Portal and The Cancer Genome Atlas (TCGA), were used to validate our results. Oral cancer tissues and five oral cancer cell lines (SCC-14, SAS, CA9-22, HSC-3, and OECM-1) were used to investigate the correlations of *ALDH7A1* rs13182402 polymorphisms and ALDH7A1 expression levels. Based on this study, we discovered the impact and functions of *ALDH7A1* polymorphism in oral cancer.

## RESULTS

### Baseline characteristics

A total of 1332 patients with oral cancer and 1191 healthy controls were enrolled. All the participants were male. No major age difference between the patients and healthy controls was observed (*P* = 0.920). Due to the observational study, patients with oral cancer were significantly more likely to smoke cigarettes, drink alcohol, and chew betel quid than the healthy controls were (all *P* < 0.001). The basic characteristics of the participants are presented in Table 1.

**Table 1:** Basic characteristics of the patients with oral cancer and healthy controls.

**Variable**	**Patients (*N* = 1332)**	**Controls (*N* = 1191)**	***P* value**
Age (yrs)			*P* = 0.476
≧55	705 (52.9%)	628 (52.7%)
<55	627 (47.1%)	563 (47.3%)
Cigarette smoking			P < 0.001^*^
Yes	1124 (84.4%)	632 (53.1%)
No	208 (15.6%)	559 (46.9%)
Alcohol drinking			*P* < 0.001^*^
Yes	635 (47.7%)	236 (19.8%)
No	697 (52.3%)	955 (80.2%)
Betel quid chewing			*P* < 0.001^*^
Yes	994 (74.6%)	199 (16.7%)
No	338 (25.4%)	992 (83.3%)
Pathologic staging			
I + II	622 (46.7%)		
III + IV	710 (53.3%)		
Pathologic T staging			
T1 + T2	667 (50.1%)		
T3 + T4	665 (49.9%)		
Pathologic N staging			
N0	876 (65.8%)		
N+	456 (34.2%)		
Pathologic M staging			
M0	1322 (99.2%)		
M1	10 (0.8%)		
Histological differentiation			
Well	186 (14.0%)		
Moderate to poor	1146 (86.0%)		

### *ALDH7A1* SNPs

Two *ALDH7A1* SNPs, namely rs13182402 and rs12659017, were sequenced for all participants. Both SNPs are located on chromosome 5. The allele frequencies of the SNPs for the East Asian population are 5.75% and 70.4%, respectively, as reported by the 1000 Genomes Project. The clinical significance of these two SNPs is not reported in ClinVar (Table 2).

**Table 2:** Detailed information of *ALDH7A1* SNPs from dbSNP.

**dbSNP**	**rs13182402**	**rs12659017**
Organism	*Homo sapiens*	*Homo sapiens*
Position^1^	chr5:126582456	chr5:126652483
Nucleotide change	A>G	G>A
Variation type	SNV	SNV
Minor allele frequency^2^	G = 0.0575	A = 0.7044
Gene consequence	Intron variant	None
Clinical significance	Not reported in ClinVar	Not reported in ClinVar

^1^GRCh38.p12. ^2^Minor allele frequency using the East Asian population from 1000 Genomes.

### Influence of *ALDH7A1* SNPs in oral cancer development

The incidences of *ALDH7A1* rs13182402 and rs12659017 polymorphism between the patients with oral cancer and healthy controls were comparable. For the AORs, which were because of different basic characteristics and adjusted by age, smoke cigarettes, drink alcohol, and chew betel quid, also showed that cancer development risk between these two groups was no different. In Taiwan, oral cancer is the largest subgroup of HNSCC, and more than 80% of patients with oral cancer habitually chew betel quid [21, 22]. Betel quid chewing significantly contributes to the development of oral cancer [23–25]. Thus, the analysis classified participants into categories of alcohol drinkers and betel quid chewers. As shown in Table 3, no significant differences were observed between oral cancer patients with *ALDH7A1* rs13182402 and rs12659017 and those with the wild-type (WT) gene. Moreover, no associations were observed in the alcohol drinker or betel quid chewer (Table 3).

**Table 3:** Odds ratios (OR) and 95% confidence interval (CI) of oral cancer associated with *ALDH7A1* genotypic frequencies.

**Variable**	**Patients (*N*, %)**	**Controls (*N*, %)**	**OR (95% CI)**	**AOR (95% CI)^a^**
**All (both Betel quid chewer and non-betel quid chewer)**				
	***N* = 1332**	***N* = 1191**		
**rs13182402**				
AA	1161 (87.2%)	1063 (89.3%)	1.000 (reference)	1.000 (reference)
AG	166 (12.5%)	125 (10.5%)	1.216 (0.950–1.556)	1.177 (0.868–1.597)
GG	5 (0.4%)	3 (0.3%)	1.526 (0.364–6.401)	1.600 (0.293–8.725)
AG+GG	171 (12.8%)	128 (10.7%)	1.223 (0.959–1.561)	1.188 (0.879–1.605)
**rs12659017**				
AA	698 (52.4%)	611 (51.3%)	1.000 (reference)	1.000 (reference)
AG	534 (40.1%)	490 (41.1%)	0.954 (0.810–1.124)	1.058 (0.863–1.296)
GG	100 (7.5%)	90 (7.6%)	0.973 (0.431–1.319)	0.919 (0.634–1.333)
AG+GG	634 (47.6%)	580 (48.7%)	0.957 (0.818–1.119)	1.033 (0.851–1.253)
**Alcohol drinker**				
	***N* = 635**	***N* = 236**		
**rs13182402**				
AA	560 (88.2%)	205 (86.9%)	1.000 (reference)	1.000 (reference)
AG	73 (11.5%)	30 (12.7%)	0.891 (0.566–1.403)	0.822 (0.481–1.406)
GG	2 (0.3%)	1 (0.4%)	0.732 (0.066–8.117)	0.757 (0.046–12.557)
AG+GG	75 (11.8%)	31 (13.1%)	0.886 (0.566–1.386)	0.821 (0.484–1.391)
**rs12659017**				
AA	332 (52.3%)	122 (51.7%)	1.000 (reference)	1.000 (reference)
AG	256 (40.3%)	93 (39.4%)	1.012 (0.738–1.386)	1.154 (0.798–1.670)
GG	47 (7.4%)	21 (8.9%)	0.822 (0.472–1.432)	0.812 (0.426–1.548)
AG+GG	303 (47.7%)	114 (48.3%)	0.977 (0.724–1.317)	1.086 (0.765–1.542)
**Betel quid chewer**				
	***N* = 994**	***N* = 199**		
**rs13182402**				
AA	868 (87.3%)	174 (87.4%)	1.000 (reference)	1.000 (reference)
AG	123 (12.4%)	24 (12.1%)	1.027 (0.664–1.638)	1.024 (0.641–1.638)
GG	3 (0.3%)	1 (0.5%)	0.601 (0.062–5.815)	0.608 (0.062–5.936)
AG+GG	126 (12.7%)	25 (12.6%)	1.010 (0.638–1.599)	1.007 (0.635–1.597)
**rs12659017**				
AA	524 (52.7%)	114 (57.3%)	1.000 (reference)	1.000 (reference)
AG	394 (39.6%)	66 (33.2%)	1.299 (0.934–1.807)	1.289 (0.925–1.796)
GG	76 (7.7%)	19 (9.5%)	0.870 (0.506–1.496)	0.866 (0.502–1.494)
AG+GG	470 (47.3%)	85 (42.7%)	1.203 (0.885–1.636)	1.195 (0.878–1.628)

^a^Adjusted for the effects of age, cigarette smoking, alcohol drinking, and betel quid chewer.

### Prognostic effect of *ALDH7A1* SNPs in oral cancer

The prognostic influence of *ALDH7A1* SNPs in oral cancer was also analyzed. Among the patients with and without *ALDH7A1* rs13182402 and rs12659017 polymorphism, no differences in pathologic staging, tumor size, lymph node metastasis, distant metastasis, or histologic differentiation were observed (Tables 4 and 5). Among the patients who habitually used betel quid, however, those with *ALDH7A1* rs13182402 polymorphism had less pathologic nodal metastasis than did those with the normal allele type (AG + GG vs. AA, 24.6% vs. 34.6%, *P* = 0.016; Table 4). After adjustment for other factors, *ALDH7A1* rs13182402 represented an independent favorable prognostic factor for nodal metastasis (OR [95% confidence interval (CI)] = 0.596 [0.382–0.929], *P* = 0.022; Table 6).

**Table 4:** The distributions of demographical characteristics of ALDH7A1 rs13182402 allele mutation in oral cancer.

**Variable**	**Total (*N* = 1332)**			**Alcohol drinker (*N* = 635)**			**Betel Quid Chewers (*N* = 994)**		
	**AA (*N* = 1161)**	**AG+GG (*N* = 171)**	***P* value**	**AA (*N* = 560)**	**AG+GG (*N* = 75)**	***P* value**	**AA (*N* = 868)**	**AG+GG (*N* = 126)**	***P* value**
**Stage**									
Stage I + II	544 (46.9%)	78 (45.6%)	0.413	260(46.4%)	32(42.7%)	0.313	408 (47.0%)	62 (49.2%)	0.356
Stage III + IV	617 (53.1%)	93 (54.4%)		300(53.6%)	43(57.3%)		460 (53.0%)	64 (50.8%)	
**T staging**									
T1/2	591(50.9%)	570 (49.1%)	0.067	295(52.7%)	37(49.3%)	0.336	446 (51.4%)	60 (47.6%)	0.244
T3/4	570 (49.1%)	95 (55.6%)		265(47.3%)	38(50.7%)		422 (48.6%)	66 (52.4%)	
**N staging**									
N0	758 (65.3%)	118 (69.0%)	0.193	201(62.7%)	28(37.3%)	0.45	568 (65.4%)	95 (75.4%)	0.016
N+	403 (34.7%)	53 (31.0%)		359(64.1%)	47(62.7%)		300 (34.6%)	31 (24.6%)	
**Metastasis**									
M0	1152 (99.2%)	170 (99.4%)	0.626	554(98.9%)	74(98.7%)	0.587	861 (99.2%)	126 (100.0%)	0.386
M1	9 (0.8%)	1 (0.6%)		6(1.1%)	1(1.3%)		7 (0.8%)	0 (0.0%)	
**Cell differentiated**									
Well	161 (13.9%)	25 (14.6%)	0.433	80(14.3%)	11(14.7%)	0.522	131 (15.1%)	20 (15.9%)	0.453
Moderate or poor	1000 (86.1%)	146 (85.4%)		480(85.7%)	64(85.3%)		737 (84.9%)	106 (84.1%)	

**Table 5:** The distributions of demographical characteristics of ALDH7A1 rs12659017 allele mutation in oral cancer.

**Variable**	**Total (*N* = 1332)**			**Alcohol drinker (*N* = 635)**			**Betel Quid Chewers (*N* = 994)**		
	**AA (*N* = 698)**	**AG+GG (*N* = 634)**	***P* value**	**AA (*N* = 332)**	**AG+GG (*N* = 303)**	***P* value**	**AA (*N* = 524)**	**AG+GG (*N* = 470)**	***P* value**
**Stage**									
Stage I + II	342 (49.0%)	280 (44.2%)	0.044	154 (46.4%)	138 (45.5%)	0.447	256 (48.9%)	214 (45.5%)	0.163
Stage III + IV	356 (51.0%)	354 (55.8%)		178 (53.6%)	165 (54.5%)		268 (51.1%)	256 (54.5%)	
**T staging**									
T1/2	364 (52.1%)	303 (47.8%)	0.063	174 (52.4%)	158 (52.1%)	0.505	276 (52.7%)	230 (48.9%)	0.133
T3/4	334 (47.9%)	331 (52.2%)		158 (47.6%)	145 (47.9%)		248 (47.3%)	240 (51.1%)	
**N staging**									
N+	233 (33.4%)	223 (35.2%)	0.264	209 (63.0%)	197 (65.0%)	0.323	179 (34.2%)	152 (32.3%)	0.295
N0	465 (66.6%)	411 (64.8%)		123 (37.0%)	106 (35.0%)		345 (65.8%)	318 (67.7%)	
**Metastasis**									
M0	693 (99.3%)	629 (99.2%)	0.563	328 (98.8%)	300 (99.0%)	0.55	521 (99.4%)	466 (99.1%)	0.441
M1	5 (0.7%)	5 (0.8%)		4 (1.2%)	3 (1.0%)		3 (0.6%)	4 (0.9%)	
**Cell differentiated**									
Well	99 (14.2%)	87 (13.7%)	0.436	46 (13.9%)	45 (14.9%)	0.403	76 (14.5%)	75 (16.0%)	0.291
Moderate or poor	599 (85.8%)	547 (86.3%)		286 (86.1%)	258 (85.1%)		448 (85.5%)	395 (84.0%)	

**Table 6:** Univariate and multivariate logistic regression for neck lymph node metastasis in oral cancer patients with betel quid chewing.

**Variable**	**Univariate**		**Multivariate**	
	***P* value**	**OR (95% CI)**	***P* value**	**OR (95% CI)**
**Age (yrs)**				
≥ 55 vs. <55	0.069	0.783 (0.601–1.020)		
**Tumor T status**				
T3/4 vs. T1/2	<0.001	2.864 (2.174–3.772)	<0.001	3.100 (2.337–4.113)
**Metastasis**				
M1 vs. M0	0.021	12.222 (1.465–101.938)	0.01	18.165 (2.024–162.999)
**Cell differentiation**				
Moderately or poorly differentiated vs. well differentiated	<0.001	2.465 (1.597–3.804)	<0.001	2.877 (1.829–4.524)
**rs13182402**				
AG+GG vs. AA	0.028	0.618 (0.402–0.949)	0.022	0.596 (0.382–0.929)
**rs12659017**				
AG+GG vs. AA	0.543	0.921 (0.707–1.200)		

### *ALDH7A1* allele mutation with higher mRNA Expression

**Figure 1:**
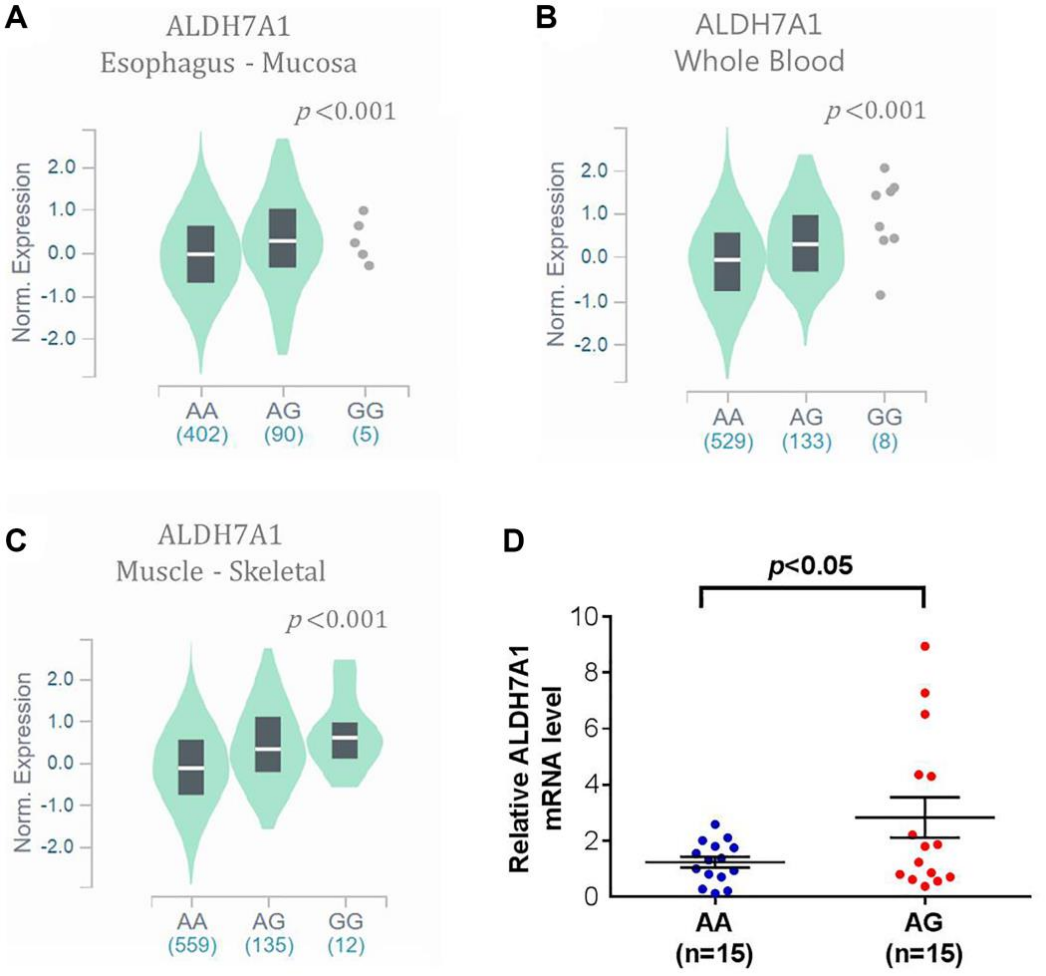
**The validated results of ALDH7A1 expression by Genotype-Tissue Expression (GTEx) Portal (https://www.gtexportal.org/home/).** (**A**–**C**) In GTEx, which enrolled 54 non-diseased tissue sites across nearly 1000 individuals, violin plots of *ALDH7A1* rs13182402 mutation was associated with higher ALDH7A1 expression level in upper aerodigestive (esophagus) mucosa, whole blood, and musculoskeletal system than those of ALDH7A1 allele normal type (*P* < 0.001, < 0.001, < 0.001, respectively). (**D**) ALDH7A1 mRNA expression in cancer tissue of 30 oral cancer patients was analyzed by quantitative real time-PCR assay.

To support our findings, some published databases were used to validate our results. In the GTEx database, which has 54 enrolled non-diseased normal tissue sites covering nearly 1000 individuals, ALDH7A1 expression in the rs13182402 mutation expression (AG + GG) was higher in upper aerodigestive (esophagus) mucosa, whole blood, and the musculoskeletal system compared with the *ALDH7A1* allele normal type (AA) (all *P* < 0.001; Figure 1A–1C). Furthermore, to realize correlation between the mRNA level of ALDH7A1 and rs13182402 polymorphism, quantitative real time-PCR (qPCR) were used to analyze ALDH7A1 mRNA level in cancer tissue of 30 oral cancer patients. We found that oral cancer patient who carry allele mutation (AG) of rs13182402 polymorphism have significantly higher mRNA levels of ALDH7A1 compare to AA genotype (Figure 1D). Taken together, these results demonstrated that *ALDH7A1* allele mutation (rs13182402) was associated with higher ALDH7A1 expression than the *ALDH7A1* SNP wild type was.

### Relationship between ALDH7A1 expression and clinical outcomes

**Figure 2:**
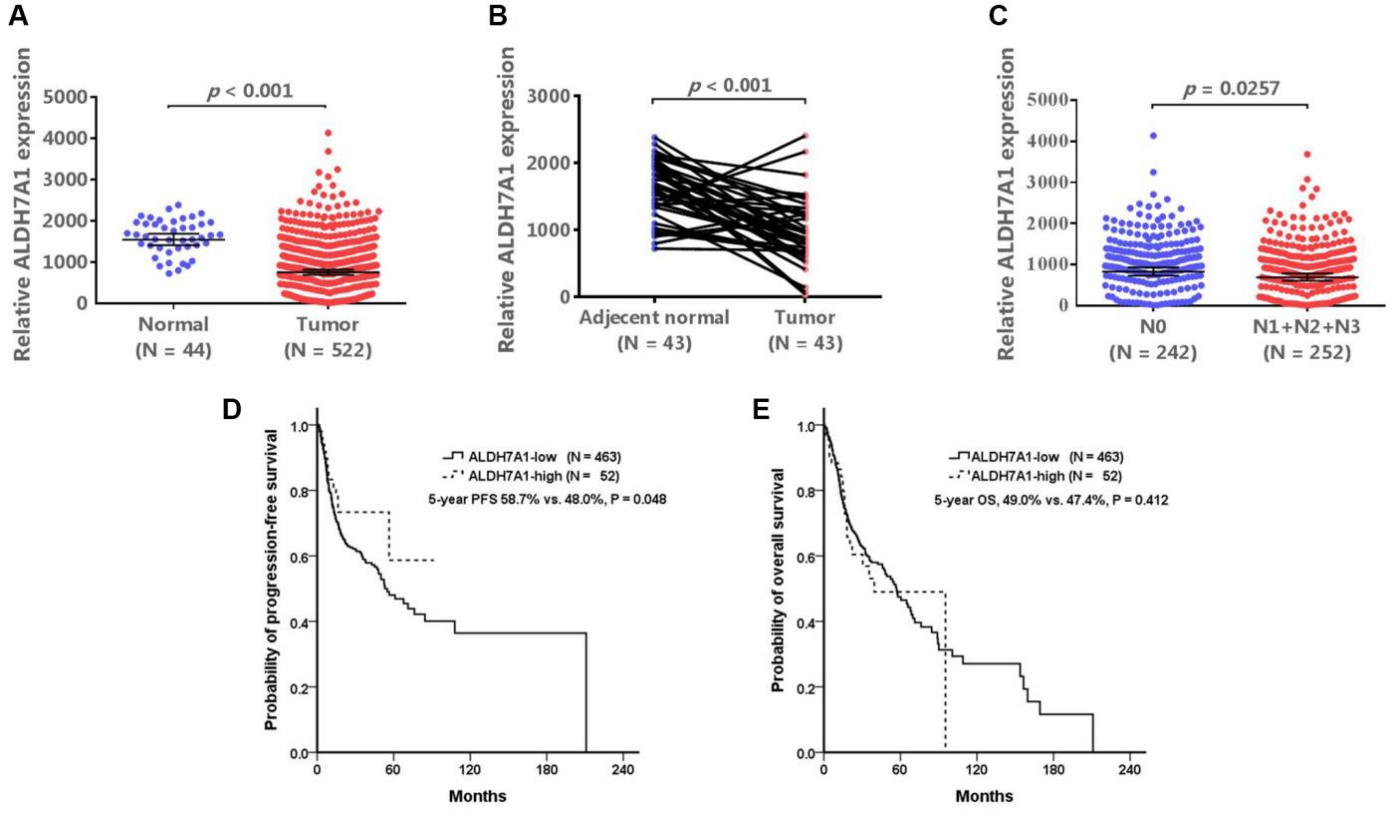
**The validated results of ALDH7A1 expression.** The Cancer Genome Atlas (TCGA) database (https://www.cbioportal.org/) was used to validate our results. (**A**, **B**) In TCGA database, ALDH7A1 expression levels were lower in tumor tissues than those in normal and adjacent normal tissues (*P* < 0.001, and < 0.001, respectively). ALDH7A1 expression levels were also lower for patients with nodal metastasis than those without nodal metastasis (*P* < 0.0257) (**C**). If the patients were divided into ALDH7A1 -high and –low groups, the high-*ALDH7A1* group tended to have better clinical outcomes than the low-*ALDH7A1* group did (5-year progression-free survival, 58.7% vs. 48.0%, *P* = 0.048; 5-year overall survival, 49.0% vs. 47.4%, *P* = 0.412) (**D** and **E**).

ALDH7A1 expression was lower in tumor tissues than in normal and adjacent normal tissues from the TCGA database (both *P* < 0.001; Figure 2A and 2B). Within the tumor tissues, ALDH7A1 expression levels were also lower for patients with nodal metastasis than for those without (*P* < 0.0257; Figure 2C). The patients could be divided into ALDH7A1-high- and ALDH7A1-low-expression groups. Because approximately 10% (12.8%, 171 out of 1332) of the patients with oral cancer had ALDH7A1 allele mutation (rs13182402), one-tenth of the patients with the highest ALDH7A1 expression in the TCGA database were classified as the high-*ALDH7A1* group, and the others were classified as the low-*ALDH7A1* group. The basic characteristics of these two groups are shown in Table 7. The high-*ALDH7A1* group tended to have better clinical outcomes than the low-*ALDH7A1* group did (5-year progression-free survival, 58.7% vs. 48.0%, *P* = 0.048; 5-year overall survival, 49.0% vs. 47.4%, *P* = 0.412) (Table 7, Figure 2D and 2E). These results indirectly demonstrate that patients with oral cancer and *ALDH7A1* rs13182402 mutation have higher ALDH7A1 expression than others do, which might result in less nodal metastasis and better prognostic outcomes.

**Table 7:** Basic characteristics of high- and low- ALDH7A1 expression patients diagnosed HNSCC from the TCGA database.

**Variable**	**High ALDH7A1 (*N* = 52)**	**Low ALDH7A1 (*N* = 463)**	***P* value**
**Age (yrs)**			
≧55	44 (69.7%)	322 (69.7%)	0.015
<55	8 (15.4%)	141 (30.3%)	
**Pathologic staging**			
I + II	12 (23.1%)	89 (19.2%)	0.759
III + IV	34 (65.4%)	310 (67.0%)	
Unknown	6 (11.5%)	64 (13.8%)	
**Pathologic T staging**			
T1 + T2	20 (38.5%)	163 (35.2%)	0.897
T3 + T4	26 (50.0%)	244 (52.7%)	
Unknown	6 (11.5%)	56 (12.1%)	
**Pathologic N staging**			
N0	21 (40.4%)	153 (33.0%)	0.527
N+	21 (40.4%)	221 (47.7%)	
Unknown	10 (19.2%)	89 (19.2%)	
**Pathologic M staging**			
M0	15 (28.8%)	168 (36.3%)	0.531
M1	0 (0.0%)	1 (0.2%)	
Unknown	37 (71.2%)	294 (63.5%)	
**Histological differentiation**			
Well	8 (15.4%)	54 (11.7%)	0.76
Moderate	28 (53.8%)	273 (59.0%)	
Poor	13 (25.0%)	110 (23.8%)	
Undifferentiated	0 (0.0%)	7 (1.5%)	
Unknown	3 (5.8%)	19 (4.1%)	
5-year progression free survival	58.70%	48.00%	0.048
5-year overall survival	49.00%	47.40%	0.412

### Functional analysis of ALDH7A1 expression in oral cancer cell lines

**Figure 3:**
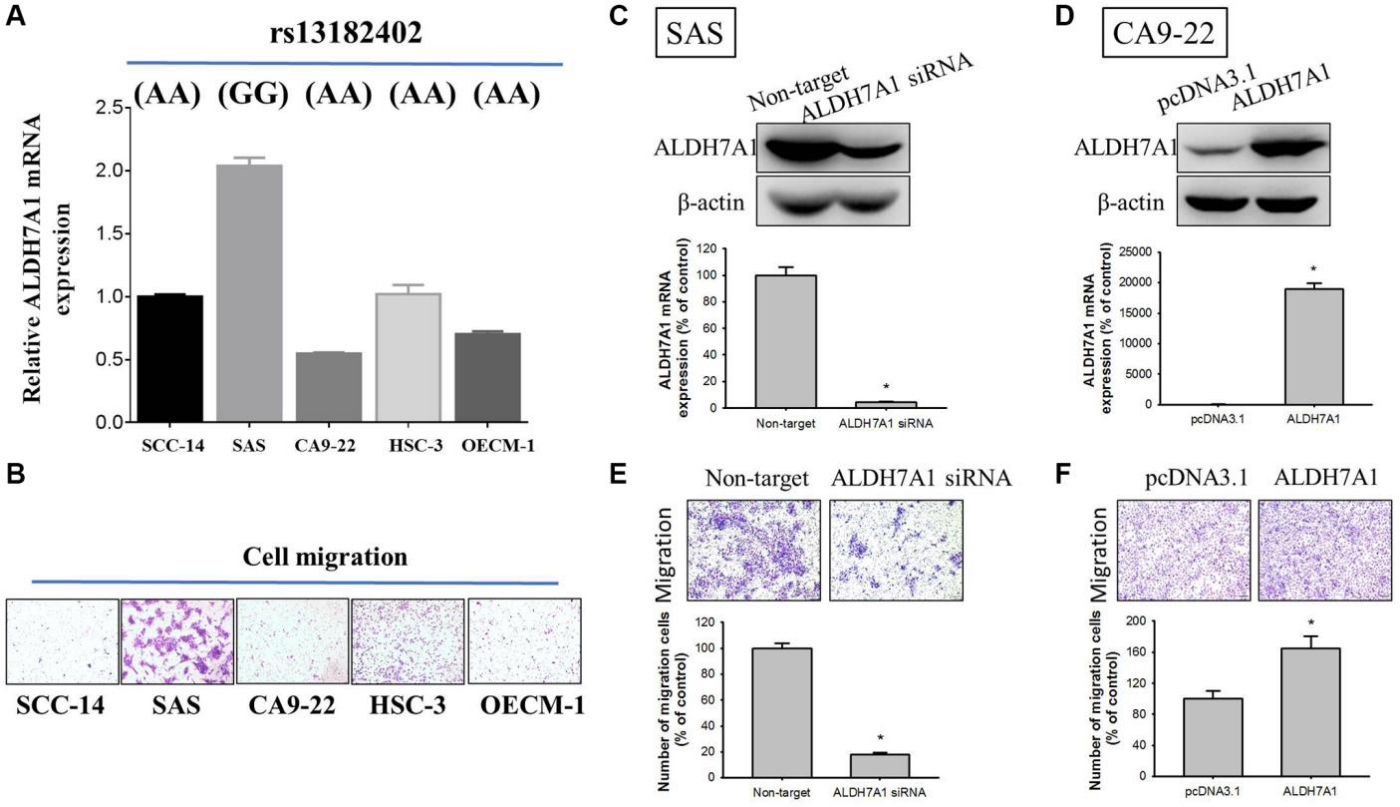
**Correlations of *ALDH7A1* rs13182402 genotypes with ALDH7A1 mRNA levels in five oral cancer cell lines.** (**A**) Upper panel, ALDH7A1 rs13182402 genotypes in oral cancer cell lines (SCC-14, SAS, CA9-22, HSC-3, and OECM-1) were detected by a TaqMan SNP Genotyping Assay. Lower panel, mRNA level of ALDH7A1 was detected by quantitative real time-PCR analysis. (**B**) The migratory ability in oral cancer cell lines (SCC-14, SAS, CA9-22, HSC-3, and OECM-1) was detected by Boyden chamber migration assays. (**C**) Western blot analysis and real time-PCR assay for ALDH7A1 protein and mRNA expressions of SAS cells after siRNA directly against the ALDH7A1 expression were conducted. (**D**) Western blot analysis and real time-PCR assay for ALDH7A1 protein and mRNA expressions of CA9-22 cells after transfection with vectors containing a constitutively active ALDH7A1 cDNA were conducted. (**E** and **F**) Boyden chamber migration assays for cell migratory ability in SAS cells and CA9-22 cells were conducted.

To further investigate correlations of *ALDH7A1* rs13182402 polymorphisms with ALDH7A1 expression levels in oral cancer, we examined rs13182402 genotypes of five oral cancer cell lines (SCC-14, SAS, CA9-22, HSC-3, and OECM-1) and found that SAS cells carried the GG genotype of rs13182402 compared to SCC-14, CA9-22, HSC-3 and OECM-1 cells which carried the AA genotype (Figure 3A, upper panel). Moreover, we detected ALDH7A1 expression by quantitative real time-PCR analysis. Among these oral cancer cell lines, we observed that SAS cells expressed higher ALDH7A1 levels than SCC-14, CA9-22, HSC-3 and OECM-1 cells (Figure 3A, lower panel). Furthermore, SAS cell lines also expressed higher migratory potential than SCC-14, CA9-22, HSC-3 and OECM-1 cells by using Boyden chamber migration assays (Figure 3B).

To determine the whether ALDH7A1 influences cellular migration, siRNA directly against the ALDH7A1 expression for SAS cells and transfection with pcDNA vector for overexpression of ALDH7A1 for CA9-22 cells was employed. We confirmed knockdown and overexpression of ALDH7A1 protein and mRNA levels through Western blotting and real time-PCR in SAS and CA9-22 cells, respectively (Figure 3C and 3D). Moreover, by using Boyden chamber migration assays, the results showed that using ALDH7A1 knockdown significantly repressed migratory potential in SAS cells (Figure 3E), whereas overexpression of ALDH7A1 significantly enhanced those potentials in CA9-22 cells (Figure 3F).

## DISCUSSION

A total of 2523 participants (1332 patients with oral cancer and 1191 healthy controls) were enrolled in this study. *ALDH7A1* polymorphisms did not influence the risk of oral cancer for all participants, alcoholic drinkers, or betel quid chewers. However, *ALDH7A1* rs13182402 represented an independent favorable prognostic factor for nodal lymph node metastasis in patients with oral cancer who chewed betel quid. Databases used in validation also indicated that the expression of *ALDH7A1* rs13182402 allele mutations was higher in upper aerodigestive (esophagus) mucosa, whole blood, and the musculoskeletal system than the expression of the *ALDH7A1* normal type was. ALDH7A1 expression in tumor cells and in patients with advanced cancer status was lower than that in normal tissue and in patients with early-stage disease. Patients with HNSCC who had high ALDH7A1 expression also tended to have superior progression-free survival outcomes compared with those having low ALDH7A1 expression. Future research to further validate these findings is warranted.

Allele frequency of *ALDH7A1* rs13182402 in the East Asian population is low. However, some points supported us to pay attention to *ALDH7A1* polymorphisms. First, based on the concept of precision medicine, although the incidences of certain genetic alterations were low, the possibility of treatment opinion for these few patients still existed, such as tropomyosin receptor kinase (TRK) inhibitors for the patients with *TRK* fusion genes, and anaplastic lymphoma kinase (ALK) inhibitor for the patients with *ALK*-mutant non-small cell lung cancer [26, 27]. Drug development for these small populations was still worth looking forward to. Besides, according to the TCGA database, the patients with high-*ALDH7A1* expression tend to have superior outcomes in progression-free survival than those with low-*ALDH7A1*. The critical issue was how to divide the patients into the high- and low- expression group. Advanced *in vivo* validations were warranted to identify the cutoff level, which might be helpful to expand the effective population. Finally, several studies reported the importance of ALDH isoenzymes in cancers [2, 13, 28, 29], and ALDH7A1 is a member of the ALDH superfamily. Based on this study, the results provided us with a better understanding of the roles of ALDH in oral cancer, especially *ALDH7A1* polymorphism.

Acetaldehyde, a metabolite from ethanol, is metabolized to acetate by ALDH, a process that results in DNA adducts, inhibited DNA repair, and DNA methylation [2]. Several studies have discussed the interaction between *ALDH* and cancer, especially within the Asian population [2, 13]. Some authors reported that ALDH genes play a role in the maintenance and differentiation of cancer stem cells [30], and others contended that high *ALDH* expression in cancer stem cells is associated with graver prognostic outcomes [31]. However, the functions of individual *ALDH* isoenzymes, such as *ALDH7A1*, have not been clearly ascertained. Wang et al. [10] revealed that *ALDH7A1* rs13182402 allele mutation reduces the development of esophageal squamous cell carcinoma (OR [95% CI] = 0.79 [0.67–0.93], *P* = 0.003). However, Lu et al. [11] suggested that *ALDH7A1* rs12659017 mutation advances colorectal cancer (OR [95% CI], 1.09 [1.06–1.12], *P* < 0.001). The different alleles on the same gene lead to potentially different influences on cancer development. In our study, both *ALDH7A1* rs13182402 and rs12659017 were not discovered to constitute risk factors for oral cancer development.

Conversely, the prognostic role of ALDH7A1 in cancer is equivocal. Giacalone et al. [32] demonstrated that in non-small-cell lung cancer, patients with higher ALDH7A1 expression on an immunohistochemical stain experienced lower recurrence-free survival than those with lower ALDH7A1 expression did. Rose et al. [33] also reported that higher ALDH7A1 expression was associated with human nodular melanoma, a melanoma subtype with a higher recurrence rate than that of superficial spreading melanoma. However, high ALDH7A1 expression can play an opposite role in other cancer types. Hoogen et al. [34] revealed that in prostate cancer, ALDH7A1 knockdown reduces intrabone growth and inhibits experimentally induced bone metastasis. Moreover, Busso-Lopes et al. [35] found that low expression of ALDH7A1 in extracellular vesicles from metastatic lymph nodes is correlated with reduced survival in oral cancer patients. Other prognosis-related mechanisms, such as the activity of peroxisome proliferator-activated receptors and DNA methylation, have also been shown to be influenced by ALDH7A1 expression [36–38]. Thus, the influence of ALDH7A1 on prognosis should be evaluated for individual cancer types.

The expression of ALDH7A1 might varies remarkably among different tissues from the published database, such as the GTEx database and TCGA database. In our study, *ALDH7A1* rs13182402 allele mutation, which was detected from the whole-blood genomic DNA, was an independent favorable prognostic factor for nodal metastasis in oral cancer. In the GTEx database, this allele mutation was validated in different non-diseased tissue sites and associated with higher ALDH7A1 expression than the normal type in blood. Moreover, oral cancer patient who carry allele mutation (AG) of rs13182402 polymorphism have significantly higher mRNA levels of ALDH7A1 compare to AA genotype. Similarly, in tumor tissue, the high-*ALDH7A1* group tended to have better progression-free survival outcomes than the low-*ALDH7A1* group did, validated by the TCGA database. And conversely, ALDH7A1 expression in advanced status (patients with nodal metastasis) was lower than that in early status (patients without nodal metastasis). The result supported that *ALDH7A1* rs13182402 allele mutation, detected from the whole-blood genomic DNA, was associated with high ALDH7A1 expression and favorable outcomes. Besides, based on our previous study, which indicated that lower *ALDH7A1* expression was associated with increased cell proliferation, DNA synthesis, and decreased apoptosis [39], several aspects warrant discussion. First, different allele mutations might result in different functions. Patients with HNSCC and mutant *ALDH7A1* (missense mutation, c.1168 G > C, rs121912707) had lower *ALDH7A1* expression than those carrying *ALDH7A1* wild-type [39], but in the current study, *ALDH7A1* rs13182402 mutation led to increased *ALDH7A1* expression. Because of the complexity of genotype-phenotype interactions and the fact that the mechanisms of epistatic interaction for different alleles of the same gene are largely unknown [40], future *in vitro* studies of individual alleles are warranted.

Betel (areca) nut, which has areca alkaloids including arecoline, arecaidine, guvacoline, and guvacine, was found to be implicated in carcinogenesis [41]. However, areca nut, the major component of betel quid, is also considered to lead to angiogenesis and cancer metastasis. Ji et al. [42] suggested that betel nut promotes massive inflammation that supports the proliferation of transforming cells. Subsequently, the vascular endothelial growth factor signaling pathway and angiogenesis are activated, causing cell growth and subsequent metastasis. Several studies have also reported that habitual betel quid chewing is associated with metabolic disorders [43–45]. However, in the TCGA database, low *ALDH7A1* expression was correlated with disorders of the metabolic-associated signaling pathways, and the cancer metastasis mechanism might arise through cancer metabolism because of *ALDH7A1* mutations [37, 39]. Nevertheless, few studies have discussed the interaction among betel quid chewing, *ALDH7A1* expression, and cancer metastasis. Future studies investigating this as well as a potential link with cancer metabolism are warranted.

Several limitations were present in this study. Although less information provided the interactions between the loci and survival outcomes in our cohort, some published databases, such as the TCGA database, indirectly remedied the impact of ALDH7A1 expression on clinical outcomes. In Taiwan, tobacco, alcohol, and betel quid chewing were reported significantly in the development of oral cancer, several studies also mentioned the impact of obesity on cancer development and prognosis. This factor would also be included in our future studies [46–48]. Advanced studies, included individual allele mutations and clinical outcomes which were corresponded to the training and validation cohorts, should be warranted in the future. Furthermore, more detailed allele information of the patients enrolled in TCGA was unavailable. Moreover, due to the complex epistatic interaction between different alleles of the same gene [40], determining whether gain or loss of function occurred in each *ALDH7A1* allele is problematic. Although patients with oral cancer who had lower ALDH7A1 expression had poorer prognoses than those with higher expression did, individual allele functions should be validated *in vitro*. Finally, few studies have discussed the interaction between betel quid chewing, ALDH7A1 expression, and cancer metastasis. Thus, more experiments in this area are also necessary.

In conclusion, this study reported that *ALDH7A1* SNPs, detected from the whole-blood genomic DNA, did not affect the risk of oral cancer. But *ALDH7A1* rs13182402 mutation was an independent favorable prognostic factor for neck lymph node metastasis in the patients who used betel quid. In addition, the published database showed that *ALDH7A1* rs13182402 mutation in whole blood coexisted with high ALDH7A1 expression. And patients with higher ALDH7A1 expression seemed to have superior prognoses than those with lower expression do. It hinted *ALDH7A1* rs13182402 mutation, associated with high ALDH7A1 expression, might be a favorable prognostic factor for patients with oral cancer. Future validations *in vitro* and *in vivo* are warranted.

## MATERIALS AND METHODS

### Study subjects

Patients diagnosed as having oral cancer at Chung Shan Medical University Hospital and Changhua Christian Hospital between 2007 and 2019 were enrolled into the case group. Moreover, healthy participants without a cancer history were enrolled from Taiwan Biobank as a control group. For the case group, all patients included were pathological diagnostic oral cancer. In Taiwan, because more than 90% of oral cancer patients were male [13, 49], females were excluded due to a rare population. The patients who were no pathological diagnosis, cytologic diagnosis only, and second primary malignancies were also excluded. Healthy participants were included between 30- to 70-year-old and had normal mental capacity. The participants who were female or diagnosed with malignancies were excluded. Clinical information, including age, pathologic staging, and any habits of chewing betel quid, smoking cigarettes, or drinking alcohol, was collected according to the medical records. All patients were staged according to the American Joint Committee on Cancer’s staging system (seventh edition) [50]. This study was approved by the Institutional Review Board of Chung Shan Medical University Hospital (CSMUH No: CS15125 and CS1-21151).

### Oral cancer cell lines and culture

The human SAS, CA9-22 and HSC-3 cell lines were purchased from and validated by the Japanese Collection of Research Bioresources Cell Bank (JCRB, Osaka, Japan). SCC-14 cells lines were purchased from were obtained from Cell Lines Service (CLS; Eppelheim, Germany). The OECM-1 cell line derived from a male Taiwanese patient [51] was maintained in RPMI-1640 medium with 10% FBS. All cells were cultured and maintained at 37°C in a 5% CO_2_ and 95% air atmosphere.

### DNA extraction and genotyping

Whole-blood specimens were collected and placed in sterile tubes containing ethylene diamine tetraacetic acid. These specimens were immediately centrifuged and then stored at −80°C. Genomic DNA was extracted from peripheral blood leukocytes by using QIAamp DNA blood mini kits (Qiagen, Valencia, CA, USA) according to previously described publication [52, 53] and then dissolved the extracts into pH 7.8 TE buffer (10 mM trisaminomethane and 1 mM ethylene diamine tetraacetic acid; pH 7.8) and then quantified by measuring the optical density at 260 nm. The final product was stored at −20°C and used as a template for polymerase chain reaction. Two *ALDH7A1* genetic polymorphism rs13182402 and rs12659017 were detected in previous study and International HapMap Project database [7]. Moreover, *ALDH7A1* rs13182402 and rs12659017 polymorphism were reported significantly in malignant diseases, such as esophageal squamous cell carcinoma, osteoporosis, and colorectal cancer [7, 10, 11]. But the roles of *ALDH7A1* polymorphisms in oral cancer were unknown. Therefore, we chose these two candidate loci in our study. Assessment of allelic discrimination for *ALDH7A1* rs13182402 (assay IDs: C__31889488_10) and rs12659017 (assay IDs: C_32255284_10) SNPs was performed using a TaqMan assay with an Applied Biosystems StepOne Real-Time Polymerase Chain Reaction System (Applied Biosystems, Foster City, CA, USA). The results were further analyzed using SDS version 3.0. The details of DNA extraction and genotyping were published in our previous study [54].

### RNA preparation and quantitative real-time PCR

Total RNA was isolated from oral cancer cell lines and oral cancer tissues using RNeasy Mini Kit (Qiagen, Valencia, CA, USA) according to previously described [55, 56]. Quantitative real-time PCR analysis was performed using TaqMan one-step PCR Master Mix (Applied Biosystems) as previously described [57].

### Cell migration assay

Five oral cancer cell lines (SCC-14, SAS, CA9-22, HSC-3, and OECM-1) cell migration was evaluated as described previously [58–61]. Briefly, after 48-hours treatment with siRNA or other manipulations, migration responses of SAS cell or other cell lines were assessed in a Boyden chamber assay with cell culture inserts of diameter 6.5-mm and pore size 8 μm (Neuro Probe) at 24-hours incubation.

### Published databases for validation

In this study, several published databases were used to validate our results. dbSNP, a public-domain archive housing a broad collection of simple genetic polymorphisms, includes the sequence context and frequency of the polymorphism [62]. GTEx Portal, a comprehensive public resource for studying tissue-specific gene expression and regulation, provides gene expression, quantitative trait loci, and histology images for nearly 1000 individuals registered at 54 non-diseased tissue sites [63]. TCGA database was downloaded from cBioPortal, an open-access resource providing more than 5000 tumor samples from 20 cancer studies [64].

### Statistical analysis

The correlations between the clinicopathological parameters were analyzed by using the Chi-square test. And Hardy Weinberg test was done to detect the population representation of genotypes of the two loci. The adjusted odds ratio (AOR)-with 95% CIs of the association between genotype frequency and oral cancer risk and clinical pathological characteristics—were measured using multiple logistic regression models after controlling for covariates. The variables with *P* values of <0.05 in univariate analyses were enrolled into the multivariate analysis. SPSS (version 21.0, SPSS Inc., Chicago, IL, USA) was used for all statistical analyses.
